# Dry fruit image dataset for machine learning applications

**DOI:** 10.1016/j.dib.2023.109325

**Published:** 2023-06-18

**Authors:** Vishal Meshram, Chetan Choudhary, Atharva Kale, Jaideep Rajput, Vidula Meshram, Amol Dhumane

**Affiliations:** a Vishwakarma Institute of Information Technology, Pune, India; b Pimpri Chinchwad College of Engineering, Pune, India

**Keywords:** Computer vision, Dehydrated fruits, Fruit Classification, Fruit detection, Image classification, Machine learning

## Abstract

Dry fruits are convenient and nutritious snacks that can provide numerous health benefits. They are packed with vitamins, minerals, and fibres, which can help improve overall health, lower cholesterol levels, and reduce the risk of heart disease. Due to their health benefits, dry fruits are an essential part of a healthy diet. In addition to health advantage, dry fruits have high commercial worth. The value of the global dry fruit market is estimated to be USD 6.2 billion in 2021 and USD 7.7 billion by 2028. The appearance of dry fruits is utilized for assessing their quality to a great extent, requiring neat, appropriately tagged, and high-quality images. Hence, this dataset is a valuable resource for the classification and recognition of dry fruits. With over 11500+ high-quality processed images representing 12 distinct classes, this dataset is a comprehensive collection of different varieties of dry fruits. The four dry fruits included in this dataset are Almonds, Cashew Nuts, Raisins, and Dried Figs (Anjeer), along with three subtypes of each. This makes it a total of 12 distinct classes of dry fruits, each with its unique features, shape, and size. The dataset will be useful for building machine learning models that can classify and recognize different types of dry fruits under different conditions, and can also be beneficial for dry fruit research, education, and medicinal purposes.

Due to their nutritional value and health advantages, dry fruits have been consumed for a very long time. One of the best strategies to improve general health is to include dry fruits in the diet.

**Specifications Table**

**Table:** 

Subject	Agriculture Engineering, Machine Learning
Specific subject area	Dry Fruit Classification and Recognition
Type of data	Images
How the data were acquired	The image for the dry fruit dataset was captured with a high-resolution smartphone camera.
Data format	Raw
Description of data collection	The high-definition mobile phone camera was used to capture the photographs of the dry fruits. The dimensions of the dry fruits’ original .heic pictures are 3024 × 4032. These images have been scaled to 512 × 512 pixels and converted to .jpg format. There are 3 classes of 4 types of dry fruit, namely, Almond Regular, Sanora, and Mamra. Cashew has Regular, Special, and Jumbo. Raisin has Black, Grade 1, and Premium. Fig has Small, Medium, and Jumbo. Hence, a total of 12 different classes are contained in the dataset. The dry fruits were taken in various lighting conditions and backgrounds namely, artificial light and natural light, while the backgrounds included white, black, green, and human palms.
Data source location	Vishwakarma Institute of Information Technology, Kapil Nagar, Kondhwa Budruk, Pune – 411 048. Maharashtra, India.
Data accessibility	Repository name: Dry Fruit Image Dataset Data identification number: 10.17632/yfhgn8py5f.1 Direct URL to data: https://data.mendeley.com/datasets/yfhgn8py5f

## Value of the Data

• Food identification: A dataset including images of many dry fruit varieties could be useful for identifying foods, such as for quality assurance in food production or consumer education.
• Machine learning: A dataset of images of dry fruits could be used to train machine learning models for tasks such as dry fruit recognition, classification, and detection.
• Marketing and advertising: A dataset of high-quality images of dry fruits could be valuable for marketing and advertising purposes, such as for use in product catalogues, advertisements, and packaging design.
• Research purposes: A dataset of images of dry fruits could be valuable for research purposes, such as studying the morphological and structural characteristics of different types of dry fruits.

## 1 Objective

High-quality image dataset of major dry fruits under different lighting conditions and background will be useful for training machine learning models and assessing their efficacy in tasks like image classification and quality assessment. The created dry fruit dataset will aid in building machine learning models to automate operations like sorting and quality control in the dry fruit business in real time, which will be helpful to the stakeholders i.e., food industries, wholesalers, and end consumers.

## 2 Data Description

**Fig. 1:**
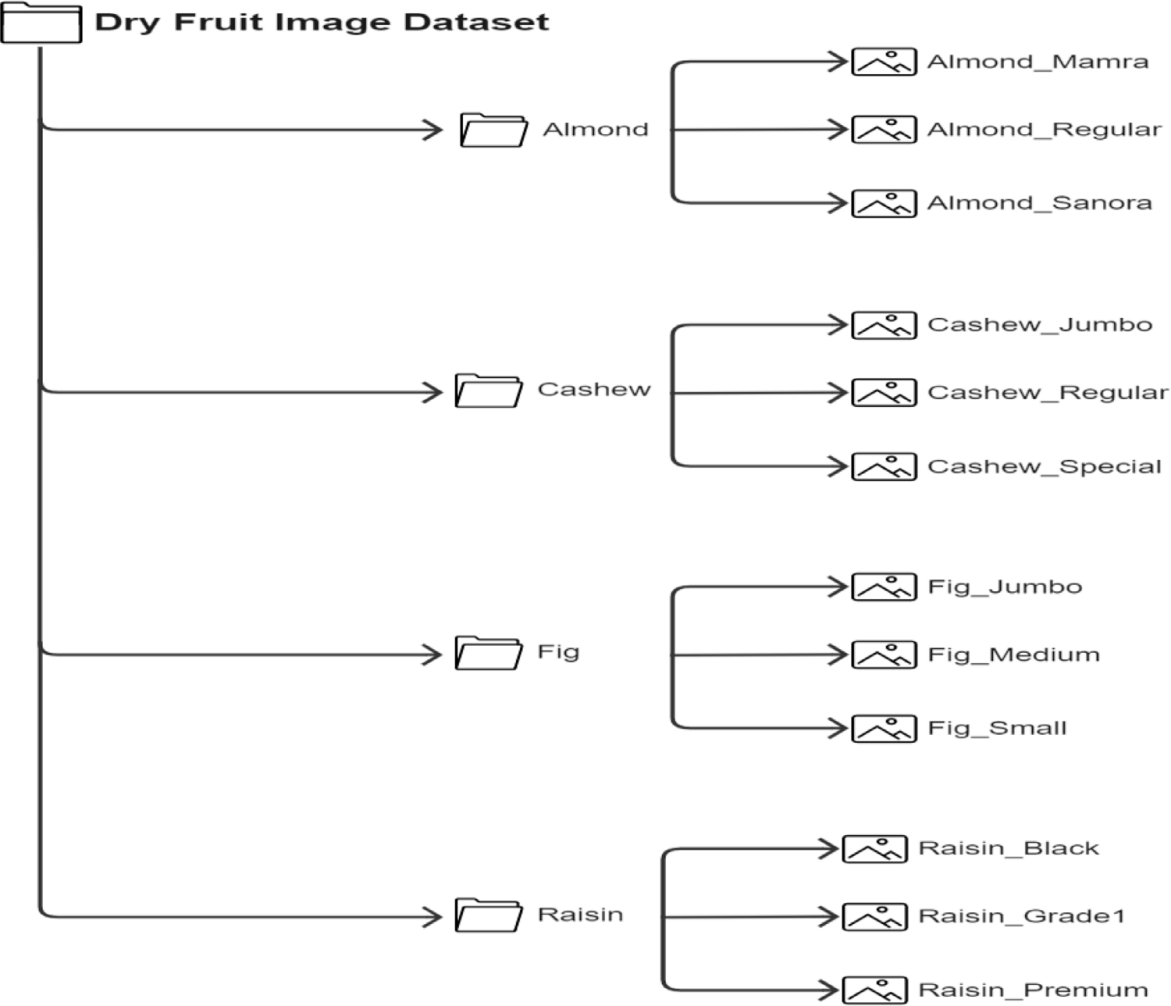
Dry fruit dataset directory structure.

**Fig. 2:**
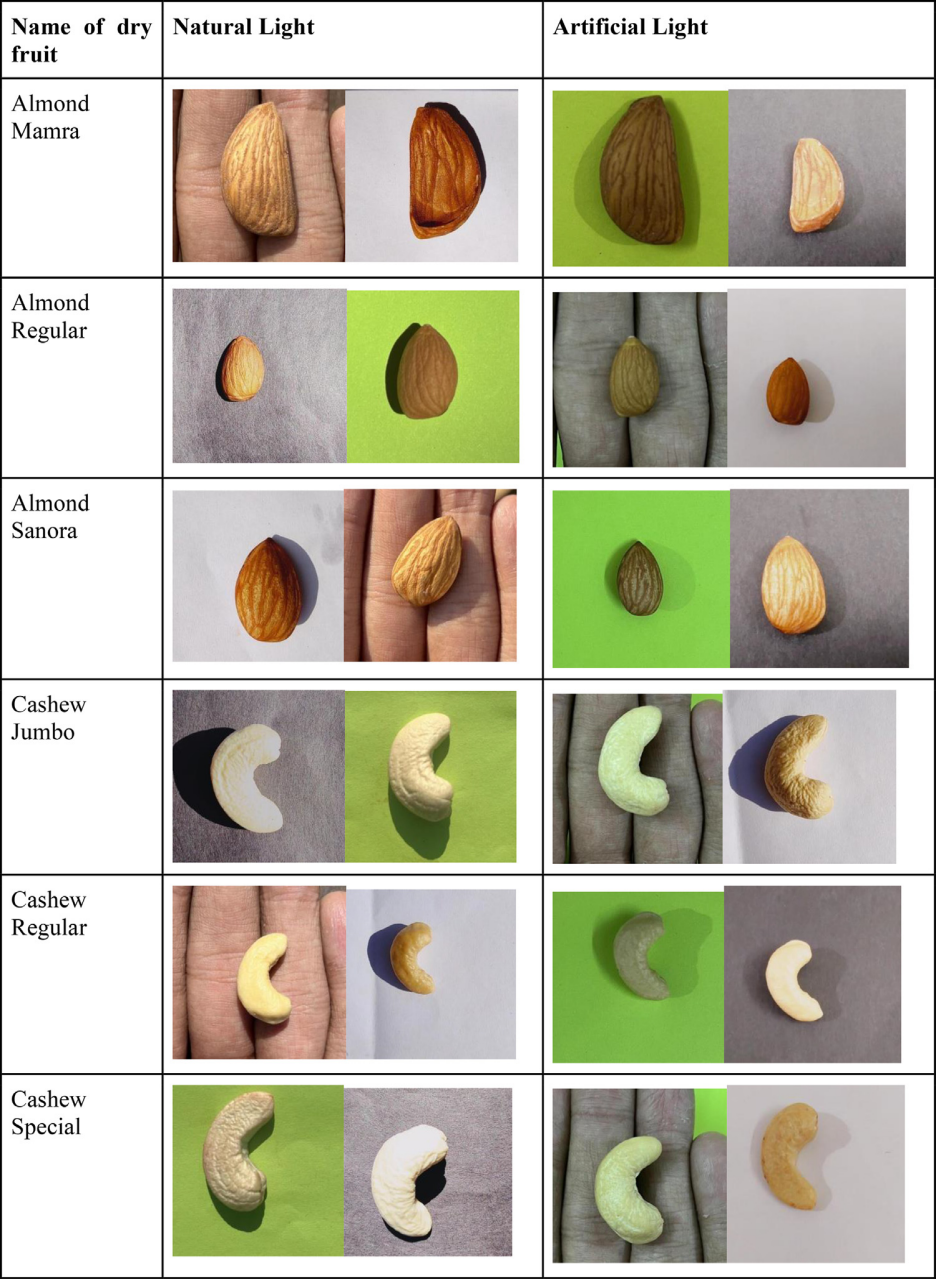

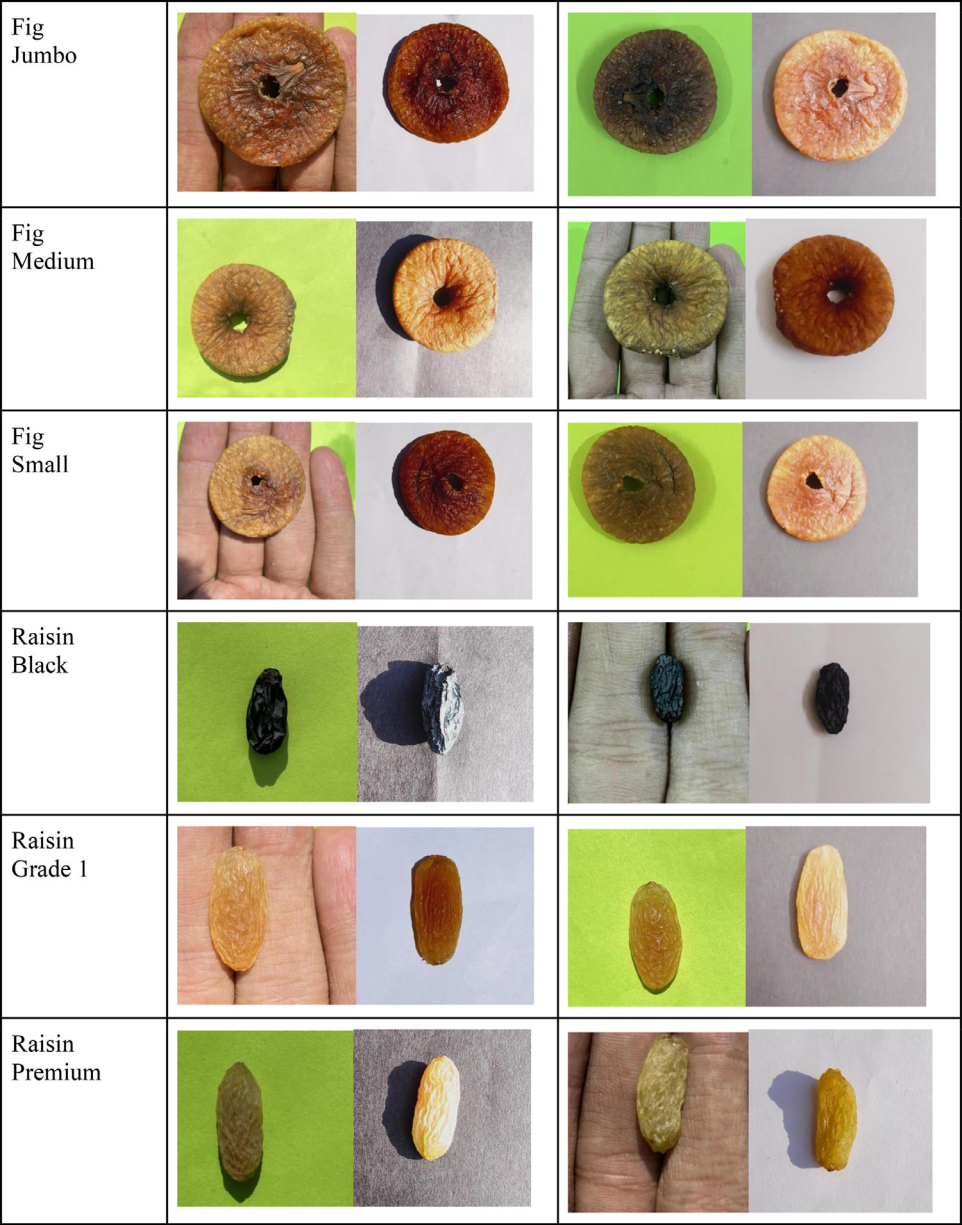
Sample Images of the dataset.

The Dry Fruit Image Dataset was created to include high-quality images of major dry fruits that are consumed and exported. It consists of four types of dry fruit each, namely, Almond, Cashew, Dried Fig, and Raisins. Each type of dry fruit is further categorized into three major subclasses. Almond has three subclasses namely, Regular, Sanora, and Mamra. Cashew has subclasses namely, Regular, Special, and Jumbo. Raisin has subclasses namely, Black, Grade 1, and Premium. Fig has subclasses namely, Small, Medium, and Jumbo. Hence, a total of 12 different classes are contained in the dataset. Fig. 1 describes the detailed directory structure of the dataset. The dry fruits were taken in various lighting conditions and backgrounds, namely, artificial light and natural light, while the backgrounds included white, black, green, and human palms. The look of dry fruits, which also impacts their marketability, can be used to judge their quality to a large extent [4]. Although there are many datasets on fruits and vegetables, those working on machine learning models and/or apps need a dataset on dry fruits due to the numerous health benefits they offer [1,2,3,10]. Machine learning models can correctly categorize and identify the type of data when a decent dataset is available [5,6,7,8,11,12]. The dataset is used for cutting-edge dry fruit-related research, education, and medical applications such as spotting fungal infections in dry fruit [9]. Fig 1. Depicts the directory structure of the Dry Fruit Image Dataset, and a few sample images from the dataset are shown in Fig. 2.

## 3 Experimental Design, Materials and Methods

### 3.1 Experimental design

**Fig. 3:**
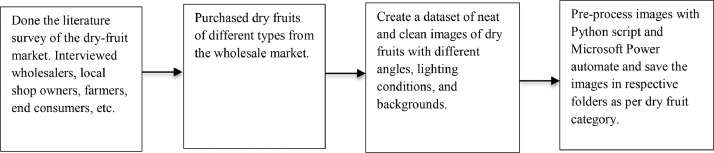
Data acquisition process of dry fruit.

The process of data acquisition for dry fruits is presented in Fig 3. The dry fruit images were captured using two different makes of camera, that were Apple's iPhone 13 and Motorola's Moto G40 fusion mobiles' rear camera having high resolution. In all, 11500+ images were captured with a camera and then stored in various folders according to their category and classification.

Four different backgrounds, two lighting conditions, and various angles are used for capturing the images of dry fruit. Images were pre-processed using a Python script and Microsoft Power Automate. The dimensions of the images, 512 × 512 make it easier to build object classification models. Table 1 describes the data acquisition steps. Dry fruits were purchased from the wholesale market of PUNE, INDIA from February to March. Dataset creation and pre-processing are done in April.

**Table 1:** Steps of data acquisition.

Sr. no.	Step	Duration	Activity
1	Collection of Data	February to March	The images of dry fruit images were captured in natural and artificial light from different angles and different backgrounds.
2	Performing pre-processing on collected data and final Dataset creation	April	The Original images were reduced to 512 × 512 dimensions using Python script. Further, as per the category of images, they were segregated into the respective folders

## 4 Materials or Specifications of the Image Acquisition System

Images of the dry fruit were taken using an Apple iPhone 13 with a 12-megapixel back camera and a Motorola Moto G40 Fusion with a 64-megapixel rear camera. Using a Python script, all of the dataset's original 3024 × 4032 image files were resized to 512 × 512 dimensions. The pictures have.jpg extensions. The photographs are taken under a range of environmental circumstances, including various lighting situations and backgrounds shot from various viewpoints.

All of the images were arranged in the following order: almond, cashew, fig, and sultana. There are three separate folders for each category/grade of dry fruit, such as Mamra, Sanora, Regular for Almond, and so on.

## 5 Method

After the survey in the local stores and wholesaler market, all twelve classes of dry fruits i.e. Almond Mamra, Almond Regular, Almond Sanora, Cashew Jumbo, Cashew Regular, Cashew Special, Fig Jumbo, Fig Medium, Fig Small, Raisin Black, Raisin Grade 1, Raisin Premium were purchased from PUNE, INDIA. Data collection took place in February and March. In the VIIT lab, typical images were taken in a variety of lighting, background, and angle situations. The dataset utilized in this study comprises two primary light sources: Natural Sunlight and Artificial light. Natural Sunlight served as the natural light source, with a range of sunlight angles spanning from 60° to 120°. Additionally, two LEDs were employed as the Artificial light sources. These LEDs were positioned at a 45° angle relative to the surface of the background setup, one on each side. For further reference, the table provided below presents the detailed specifications of the Artificial LED light sources. Using a Python script, all the original photos, which were all 3042 × 4032 in size, were shrunk to 512 × 512 and then given new names using the Microsoft Power Automate tool. Table 2 describes the classes, number of images taken, lighting conditions, and background in which the images are taken in detail and Table 3 describes the specifications of the artificial light setup.

**Table 2:** Dry fruit dataset details.

Dry Fruit	Dry Fruit Category	Image capture direction	Various backgrounds and lightning conditions for image capture	Image count for each category	Image Total
Almond	Mamra, Regular, Sanora	Front Direction, Back Direction, Side Direction, Side Direction, Top Direction, Bottom Direction	Lighting condition: Sunlight, Artificial Light Backgrounds: Black, White, Green, and Human Palm	960	2880
Cashew	Jumbo, Regular, Special	Front Direction, Back Direction, Side Direction, Side Direction, Top Direction, Bottom Direction	Lighting condition: Sunlight, Artificial Light Backgrounds: Black, White, Green, and Human Palm	960	2880
Fig	Jumbo, Medium, Small	Front Direction, Back Direction, Side Direction, Side Direction, Top Direction, Bottom Direction	Lighting condition: Sunlight, Artificial Light Backgrounds: Black, White, Green, and Human Palm	960	2880
Raisin	Black, Grade 1, Premium	Front Direction, Back Direction, Side Direction, Side Direction, Top Direction, Bottom Direction	Lighting condition: Sunlight, Artificial Light Backgrounds: Black, White, Green, and Human Palm	960	2880

Total					11520

**Table 3:** Artificial light specification.

Sr. No.	Parameter	Value
1	Brand	PHILIPS
2	Light Type	LED
3	Wattage	20 Watts
4	Light Colour	White
5	Quantity	2
6	Angle of Incidence	45°
7	Colour Temperature	6500 Kelvin
8	Brightness	2000 Lumen
9	Model Name	Astra Line
10	Indoor/Outdoor Usage	Outdoor, Indoor
11	Power Source	Corded Electric
12	Height	10 Centimetres
13	Length	10 Centimetres
14	Width	10 Centimetres
15	Weight	200 Grams
16	Manufacturer	Signify Innovations India Limited
17	Country of Origin	India

## Ethics Statements

Experiments on humans or animals are not involved in this study.

## CRediT Author Statement

**Vishal Meshram:** Conceptualization, Project administration; **Vidula Meshram:** Supervision, Writing – review & editing; **Chetan Choudhary:** Writing – original draft, Data curation; **Atharva Kale:** Investigation, Data curation; **Jaideep Rajput:** Data curation, Formal analysis; **Amol Dhumane:** Metodology, Resources.

## Declaration of Competing Interest

The authors declare that they have no known competing financial interests or personal relationships that could have appeared to influence the work reported in this paper.

## Data Availability

Dry Fruit Image Dataset (Original data) (Mendeley Data).
